# Engineering Inorganic Nanoparticles to Induce Cuproptosis: A New Strategy for Cancer Therapy

**DOI:** 10.3390/pharmaceutics17111383

**Published:** 2025-10-24

**Authors:** Zhenxing Jiang, Jianwei Dai, Juanjuan Jiang, Shenghe Deng, Junnan Gu, Jun Wang, Mian Chen, Wentai Cai, Ke Wu, Kaixiong Tao, Ke Liu, Kailin Cai

**Affiliations:** 1 Department of Gastrointestinal Surgery, Union Hospital, Tongji Medical College, Huazhong University of Science and Technology, Wuhan 430022, China; d202482252@hust.edu.cn (Z.J.); daijianwei@hust.edu.cn (J.D.); junwangwhuh@hust.edu.cn (J.W.); d202381980@hust.edu.cn (M.C.); wuke201288@hust.edu.cn (K.W.); kaixiongtao@hust.edu.cn (K.T.); 2 School of Medicine and Health Management, Tongji Medical College, Huazhong University of Science and Technology, Wuhan 430022, China; jiangjuanjuan@hust.edu.cn; 3 Medical Academic Affairs Office, Tongji Medical College, Huazhong University of Science and Technology, Wuhan 430022, China; 4 Center for Liver Transplantation, Union Hospital, Tongji Medical College, Huazhong University of Science and Technology, Wuhan 430022, China; dengshenghe@hust.edu.cn; 5 Department of Thoracic Surgery, Union Hospital, Tongji Medical College, Huazhong University of Science and Technology, Wuhan 430022, China; gujunnan@hust.edu.cn; 6 Department of Critical Care Medicine, Union Hospital, Tongji Medical College, Huazhong University of Science and Technology, Wuhan 430022, China; m202576503@hust.edu.cn

**Keywords:** inorganic nanoparticles, cuproptosis, drug delivery, tumor therapy

## Abstract

Cuproptosis is a newly identified type of copper (Cu)-dependent programmed cell death (PCD), triggered when Cu directly interacts with the lipoylated components of the tricarboxylic acid (TCA) cycle, and it has shown significant antitumor potential. However, challenges such as insufficient Cu accumulation in tumor cells, systemic toxicity, and the lack of specific carriers for effectively inducing cuproptosis hinder its practical application. Inorganic nanoparticles (INPs) present a promising solution due to their unique ability to target specific areas, potential for multifunctional modification, and controlled release capabilities. Their distinctive physicochemical properties also enable the integration of synergistic multimodal cancer therapies. Therefore, utilizing INPs to induce cuproptosis represents a promising strategy for cancer treatment. This review systematically elucidates the regulatory mechanisms of Cu homeostasis and the molecular pathways underlying cuproptosis, thoroughly discusses current INP-based strategies designed to trigger cuproptosis, and comprehensively examines the multi-modal synergistic antitumor mechanisms based on cuproptosis. Finally, we also address the current challenges and future perspectives in developing clinically applicable nanoplatforms aimed at harnessing cuproptosis for effective cancer therapy.

## 1. Introduction

Cu is an essential trace element in living organisms, playing a crucial catalytic and regulatory role in various physiological processes [1,2]. As a cofactor for essential metalloproteins, including cytochrome c oxidase (COX), superoxide dismutase (SOD), and tyrosinase, Cu plays an important role in electron transfer via the Cu^2+^/Cu^+^ reversible redox reaction. This process directly regulates mitochondrial respiratory chain function, facilitates the clearance of reactive oxygen species (ROS), and contributes to melanin synthesis [3,4,5,6]. Cu also plays an essential role in metabolic processes of human organs. An imbalance in levels of Cu can result in toxicity affecting multiple organs and systemic functions, as observed in Wilson’s disease [7]. Furthermore, Cu accumulation has been observed in the serum of patients with Huntington’s disease [8], Alzheimer’s disease [9], and atherosclerosis [10]. To maintain Cu homeostasis, organisms have evolved a sophisticated regulatory network that includes Cu transporters, Cu chaperones, and metallothioneins. These components work together to tightly regulate the concentration of Cu in the cytoplasm [11,12]. Cu can also function as a signaling molecule promoting tumor cell proliferation, invasion, and angiogenesis by activating or inhibiting signaling pathways such as phosphatidyqinositol-3 kinase/protein kinase B, hypoxia inducible factor 1 subunit alpha (HIF-1α), and nuclear factor kappa-B [13,14,15,16,17].

The phenomenon of cell death induced by Cu was first discovered in the 1980s. However, its mechanism remained unclear for an extended period [18]. In March 2022, Tsvetkov’s team published a landmark study which for the first time clarified that cuproptosis is a novel form of PCD, distinct from known modes of cell death as ferroptosis, apoptosis, pyroptosis, and autophagy [19]. Cuproptosis displays unique molecular characteristics, as Cu directly targets acetylated TCA cycle proteins in the mitochondria. This interaction leads to the abnormal aggregation of these metabolic enzymes and a concomitant loss of iron-sulfur cluster proteins [19]. The Cu ionophores, such as elesclomol (Es) and disulfiram (DSF), play a crucial role in elucidating the mechanism of cuproptosis by specifically facilitating the transport and accumulation of Cu within cells and mitochondria [20,21]. Utilizing the Cu to induce cuproptosis has emerged as a highly promising antitumor strategy [22,23].

Nevertheless, the application of cuproptosis in cancer treatment still faces challenges. Firstly, Cu has difficulty accumulating effectively in tumor cells, which hinders its ability to achieve therapeutic effects on tumors [24]. Secondly, while Cu ionophores can enhance the concentration of Cu within cells, they encounter challenges such as poor water solubility, insufficient chemical stability, and concerns related to biological safety [12]. Thirdly, excessive Cu is cytotoxic, as its accumulation in non-target organs can lead to systemic toxicity. Additionally, physiological barriers within tumor microenvironment (TME) impact the efficiency of drug accumulation. Finally, tumor cells that rely on glycolysis exhibit low sensitivity to cuproptosis [19,25]. Overall, the induction of cuproptosis by Cu ionophores faces challenges related to low efficiency and poor safety. However, advances in nanomedicine present promising solutions to enhance cuproptosis. Nanoparticles can improve drug delivery efficiency, enhance the solubility of hydrophobic drugs, and thereby increase their bioavailability. Furthermore, after surface modification, nanoparticles can achieve tumor-specific accumulation through either active or passive targeting. INPs are key components of nanomaterials, possessing unique physicochemical properties. They not only exhibit a high drug loading capacity but can also integrate physical therapies such as photothermal therapy (PTT) and magnetic hyperthermia, thereby providing new strategies for the combination of cancer therapy [26,27].

This review begins by outlining Cu metabolism pathways and their regulatory networks, followed by an in-depth discussion of the molecular mechanisms of cuproptosis. It then introduces the feasibility of targeting cuproptosis for cancer treatment and systematically summarizes various strategies based on INPs to induce and enhance cuproptosis, including improving Cu delivery efficiency, promoting Cu^+^ generation, reducing Cu^2+^ chelation, and implementing metabolic reprogramming. Furthermore, it elaborates on the synergistic effects between cuproptosis and multimodal therapies such as PTT, sonodynamic therapy, immunotherapy (IT), and radiotherapy (RT). Finally, the review discusses current challenges in the field and explores future directions for developing clinically translatable nanoplatforms to promote the effective application of cuproptosis in cancer therapy. Currently, there are relatively few comprehensive reviews focusing on cuproptosis induction by INPs. The novelty of this review lies in its detailed exploration of engineered INP-based strategies and mechanisms for precise regulation of cuproptosis, highlighting their potential in targeted induction of cuproptosis and synergistic multimodal therapies. In summary, INPs offer a transformative strategy to overcome delivery and targeting challenges in cuproptosis induction and enable effective integration with various anticancer therapies.

### 1.1. Cu Homeostasis

**Figure 1:**
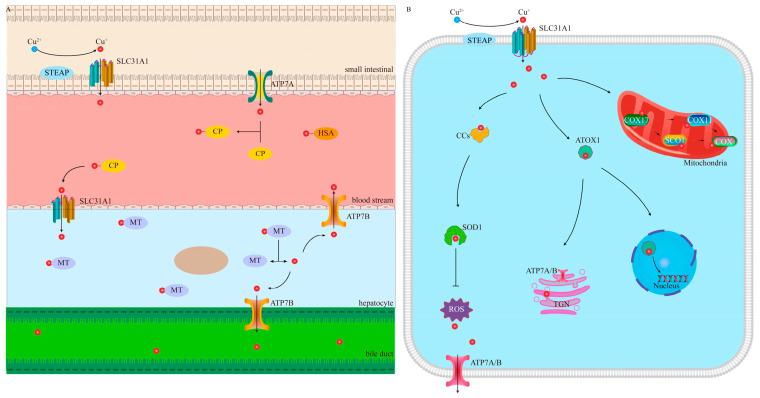
Systemic regulation and cellular metabolic pathways of Cu. (**A**) Absorption, storage, transport, and excretion of Cu. (**B**) Overview of the mediating pathways of cellular Cu metabolism. STEAP, six-segment transmembrane epithelial antigen of prostate; SLC31A1, solute carrier family 31 member 1; ATP7A, ATPase Cu Transporting Alpha; CP, ceruloplasmin; HSA, human serum albumin; MT, metallothioneins; ATP7B, ATPase Cu Transporting Beta.

Cu, an essential transition metal, plays a dual role in cellular physiology, exhibiting a “double-edged sword” effect. The intracellular concentration of Cu is maintained within a relatively low range, and even a moderate increase can lead to cellular toxicity and potentially cell death. Therefore, the uptake, distribution, and elimination of Cu are strictly regulated [4]. In normal cells, the total intracellular Cu concentration typically ranges from 10^−5^ to 10^−4^ mol/L, whereas the concentration of free Cu is extremely low, usually less than 10^−18^ mol/L, with an average of only about one free Cu per cell [28]. Compared with normal cells, tumor tissues exhibit a higher demand for Cu [4]. For example, in oral cancer [29], breast cancer [30], pancreatic cancer [31], and prostate cancer [32], Cu levels in tumor tissues and serum are generally elevated and associated with poor prognosis [33]. Cu is primarily absorbed from food in the small intestine of the human body [34]. The small intestinal epithelium absorbs Cu through Cu transporter 1. It is then transferred to the opposite side of the epithelium by the antioxidant 1 Cu chaperone and subsequently pumped into the bloodstream via ATPase Cu Transporting Alpha (ATP7A) [35]. Cu in the blood is primarily transported bound to proteins, with approximately 75% bound to ceruloplasmin and 25% bound to human serum albumin [36]. Cu is transported to the liver via the portal system, which is the primary organ for storage and excretion [37]. The liver stores Cu through metallothioneins and excretes excess Cu into bile duct via the ATPase Cu Transporting Beta (ATP7B) [38]. The absorption, storage, transport, and excretion of Cu are shown in Figure 1A.

At the cellular level, extracellular Cu^2+^ is reduced to Cu^+^ by six-segment transmembrane epithelial antigen of prostate (STEAP) and subsequently transported into the cell via solute carrier family 31 member 1 (SLC31A1) [39]. The STEAP family includes STEAP1, STEAP2, STEAP3, and STEAP4, which are involved in various biological processes [40]. Although STEAP expression is very high in prostate tissue, these proteins are also expressed in female reproductive organs such as the ovaries, uterus, and fallopian tubes [40,41]. Furthermore, studies have shown that STEAP1 and STEAP2 are overexpressed in various human cancers, including not only prostate cancer but also bladder, colon, pancreatic, ovarian, testicular, breast, and cervical cancers, as well as Ewing’s sarcoma [42,43,44]. It is noteworthy that there is no direct evidence indicating sex-specific differences in STEAP-mediated Cu utilization. Subsequently, various Cu chaperones transport Cu to specific cellular compartments, thereby maintaining Cu homeostasis while performing their respective functions. For instance, the Cu chaperone for SOD1 in the cytoplasm delivers Cu to SOD1, facilitating the elimination of ROS [45,46]. Additionally, mitochondrial chaperones play a crucial role in the assembly of COX [47]. Specifically, Cu^+^ is transferred from COX17 to synthesize cytochrome c oxidase 1 (SCO1) or COX11, which serve as Cu donors for the CuA and CuB sites of COX, respectively [48]. Meanwhile, in the nucleus, Cu can bind to transcription factors and modulate gene expression [4]. ATP7A and ATP7B are the primary Cu efflux proteins and play a crucial role in maintaining Cu homeostasis. When the intracellular concentration of Cu is at normal levels, these transport proteins are located in the trans-golgi network (TGN), where they transport Cu into the TGN to facilitate the synthesis of Cu-dependent enzymes [49]. During Cu overload, these transport proteins relocate from the TGN to vesicular compartments, where they fuse with the plasma membrane to expel excess Cu. Once Cu levels return to physiological ranges, the proteins are recycled back to the TGN [50]. The Cu metabolism pathway is shown in Figure 1B. In addition, there are proteins within the cell that bind to and store Cu. Metallothioneins and glutathione (GSH) also function as natural Cu chelators, preventing cellular damage by binding to Cu [51]. Therefore, the normal function of Cu relies on the coordinated action of Cu transporters, chaperones, and storage proteins, which collectively maintain intracellular Cu homeostasis. Experimental studies further illustrate the toxic potential of Cu overload. Tsvetkov et al. measured Cu levels in ABC1 cells using inductively coupled plasma–mass spectrometry (ICP-MS). They treated cells with Es at low concentrations (as low as 40 nM) for 2 h, which increased intracellular Cu levels by 15–60-fold (approximately 60 ng per million cells) and induced cuproptosis within 24 h [19].

Cu homeostasis is closely related to cancer biology, and its dysregulation is not merely a bystander but a key driving factor in tumor progression. In many malignant tumors, intracellular Cu levels are typically elevated. Serum Cu levels are elevated in cancer patients, typically reaching 1.5–3 times those observed in healthy individuals [52]. For instance, in breast cancer, mean serum Cu levels rise from approximately 115 μg/dL to 131 μg/dL [53], while in colorectal cancer, they increase from around 99 μg/dL to 165 μg/dL [33]. A positive correlation between Cu concentration and disease progression has also been noted. Patients with advanced lung cancer exhibit higher serum Cu levels (~150 μg/dL) compared to those in early stages (~125 μg/dL) [54]. Furthermore, Cu accumulation is even more substantial at the tissue level. Marked elevations are evident in breast cancer tissues (~21.0 μg/g versus ~9.3 μg/g in normal tissue) [55] and in leukemia cells (~52 μg/106 cells compared to ~15 μg/106 cells in normal counterparts) [56]. This disruption of Cu homeostasis transforms Cu into a potent signaling molecule that promotes carcinogenesis. Specifically, Cu serves as an essential cofactor for several key enzymes, which then promote critical processes in tumor development such as energy metabolism, ROS production, and extracellular matrix remodeling [57,58]. Moreover, the disruption of Cu homeostasis enhances cancer invasion and metastasis. It induces epithelial–mesenchymal transition and modulates the activity of lysyl oxidase, an enzyme crucial for remodeling the extracellular matrix to support metastatic spread [59]. Cu also enhances the invasiveness and angiogenesis of tumors by upregulating the expression of metalloproteinases and vascular endothelial growth factor, thereby promoting invasion and the formation of new blood vessels in the tumor microenvironment [60,61]. The critical link between Cu homeostasis and cancer is perhaps further illustrated by the frequent overexpression of ATP7A and ATP7B in invasive and drug-resistant cancers. This adaptive response highlights how cancer cells utilize Cu regulation mechanisms to maintain their malignant phenotype [62]. Maintaining Cu homeostasis in the body is significant importance.

### 1.2. Molecular Mechanisms of Cuproptosis

**Figure 2:**
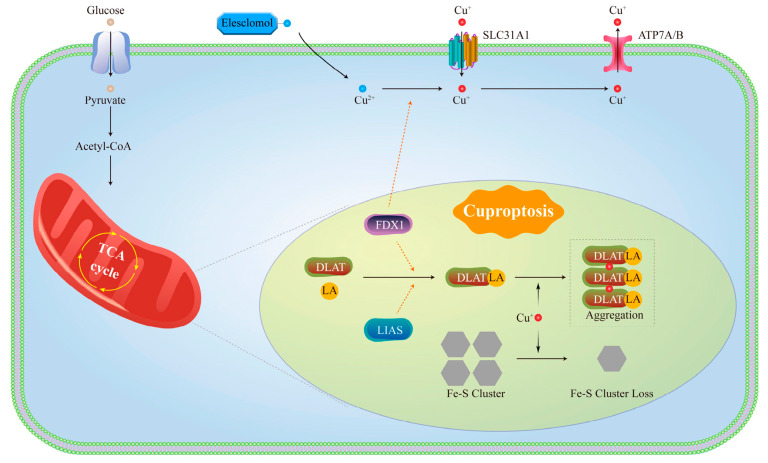
The molecular mechanisms of cuproptosis. TCA, tricarboxylic acid cycle; SLC31A1, solute carrier family 31 member 1; ATP7A/B, ATPase Cu Transporting Alpha and Beta; FDX1, ferredoxin 1; DLAT, dihydrolipoamide S-acetyltransferase; LIAS, lipoic acid synthase; LA, lipoic acid.

The relationship between Cu and PCD has garnered significant attention in recent years. Although the phenomenon of Cu-induced cell death was identified as early as the 1980s, its precise mechanism was only elucidated by Tsvetkov’s team in 2022 [19], who introduced the concept of “cuproptosis”. A notable characteristic of Cu ionophores and Cu-treated cells is the pronounced increase in ROS levels, which has long been regarded as the primary cause of cell death [63,64]. This is because ROS scavengers can mitigate the severity of cuproptosis in specific cells [65,66,67]. However, the elimination of ROS does not consistently prevent Cu-induced cell death, indicating that ROS may not be the primary factor responsible for cuproptosis [19,68]. Moreover, studies have demonstrated that antioxidants are ineffective in reversing cell damage caused by Es and Cu. In contrast, GSH and Cu chelators, such as ammonium tetrathiomolybdate, can effectively prevent cytotoxicity [19]. At the same time, Tsvetkov’s research indicates that cell death induced by Cu ionophores primarily depends on the accumulation of intracellular Cu rather than on Cu ionophores itself [19]. Furthermore, this type of cellular damage cannot be mitigated by inhibitors targeting other forms of cell death, such as apoptosis, ferroptosis, and necrosis [19]. Tsvetkov et al. found that Es can transport Cu^2+^ across the membrane into the mitochondria, where Cu^2+^ is reduced by ferredoxin 1 (FDX1) to the more toxic Cu^+^ [19]. However, excessive Cu^+^ can directly bind to lipoylated dihydrolipoamide S-acetyltransferase (DLAT), an essential component of the mitochondrial TCA, leading to DLAT aggregation and cytotoxicity [19]. DLAT is a key component of the pyruvate dehydrogenase complex (PDC) in the TCA cycle, located in the mitochondrial matrix and directly exposed to a Cu-enriched environment. Its activity depends on the lipoylation modification of specific lysine residues, where the disulfide bonds (-S-S-) in lipoic acid (LA) have a strong affinity for Cu^+^, allowing the formation of a highly stable complex [69,70]. When Cu^+^ binds to DLAT, it induces a conformational change, resulting in the formation of inactive oligomers that cannot be properly degraded. This not only causes the loss of PDC function, disrupting the TCA cycle, but also leads to protein accumulation that exceeds the proteasomal degradation capacity. Consequently, Heatshockprotein70 is excessively recruited to disaggregate the DLAT oligomers, impairing the repair of other misfolded proteins and triggering a protein toxicity stress response [19]. In addition, excessive mitochondrial Cu^+^ can destabilize iron-sulfur cluster proteins through direct metal replacement, oxidative damage, and interference with biosynthesis. First, Cys79 in iron-sulfur cluster assembly 2 is a key residue for binding all types of Fe/S clusters, while Cys144 and Cys146 are critical for the formation of the [4Fe-4S] cluster. Cu^+^ has a strong affinity for sulfur ligands. On one hand, Cu^+^ binds to the conserved cysteine residues (such as Cys53) on the Fe-S cluster assembly scaffold protein Iron-sulfur cluster assembly enzyme, physically occupying the assembly sites for [2Fe-2S] and [4Fe-4S] clusters, preventing the formation of new clusters. On the other hand, Cu^+^ can displace Fe/S clusters from glutaredoxin-5 and iron-sulfur cluster assembly proteins, hindering the normal acquisition of [2Fe-2S] clusters and leading to the release and failure of cluster assembly [71]. The coordinating sulfur atoms (S^2−^) in the [4Fe-4S] cluster have a high affinity for Cu^+^, which competes with Fe^2+^, leading to the disintegration of the Fe-S cluster structure [72]. Additionally, Cu^+^ catalyzes the generation of hydroxyl radicals (·OH), which oxidize the sulfur and iron atoms within Fe-S clusters. At higher concentrations, Cu^+^ can catalyze the production of highly destructive hydroxyl radicals through a Fenton-like reaction, attacking the sulfur ligands of Fe-S clusters. ROS further oxidizes the iron ions within the Fe-S clusters, causing the clusters to become unstable and disintegrate [73]. Iron-sulfur cluster proteins are crucial for electron transfer in oxidative phosphorylation. This destabilization can result in protein toxicity and ultimately lead to cell death [19]. The molecular mechanisms of cuproptosis are shown in Figure 2. Additionally, the primary positive regulators of cuproptosis include lipoic acid synthase (LIAS), dihydrolipoamide dehydrogenase, lipoyl transferase 1, and FDX1 from the LA pathway. Additionally, the PDC contributes to this process through DLAT, pyruvate dehydrogenase E1 subunit alpha 1, and pyruvate dehydrogenase E1 subunit beta [19]. These results also confirm the significance of FDX1 and its role in regulating mitochondrial protein lipoylation during cuproptosis.

## 2. Targeted Cuproptosis for Cancer Therapy

The cuproptosis is closely linked to mitochondrial metabolism, as metabolically active tumor cells exhibit increased sensitivity to this process. Inhibition of the mitochondrial electron transport chain or pyruvate uptake diminishes the responsiveness of tumor cells to Cu inducers [19], indicating that cuproptosis can effectively suppress the growth of cells characterized by elevated levels of aerobic respiration, such as melanoma [74] and leukemia [75]. Additionally, a heightened metabolic state of mitochondria is a characteristic of tumor cell resistance to specific drugs, including proteasome inhibitors [76], cisplatin [77], and 5-fluorouracil [78]. Therefore, targeting cuproptosis may represent a promising strategy for tumor treatment. Cu ionophores play a crucial role in the exploration of cuproptosis. The most extensively studied Cu ionophores are Es and DSF. Es is a highly lipophilic Cu-binding molecule that can chelate extracellular Cu^2+^ to form the Es-Cu^2+^ complex, facilitating the entry of Cu into cells [79,80]. DSF interacts with Cu to form the metabolite bis-diethyl dithiocarbamate Cu, which facilitates the transport of Cu across the cell membrane [81]. Although preclinical trials have demonstrated significant antitumor effects of Cu ionophores, no promising results have been observed in clinical trials [82]. This may be related to the inability to maintain elevated Cu levels in tumor cells [83]. The application of INPs can enhance the cellular accumulation of Cu, demonstrating significant clinical potential in cancer therapy. Targeted delivery, controlled release, and high drug loading can elevate the concentration of Cu, improve the efficacy of cuproptosis, and minimize the side effects associated with cancer treatment. For example, Liu et al. designed a DSF/Cu^2+^-loaded MXene nanosheet system coated with PD-1-overexpressing T cell membranes, creating a bioinspired nanoplatform with immune recognition capability [84]. The PD-1 proteins on the outer surface act like “molecular patches”, enabling specific recognition and binding to PD-L1 receptors on tumor cells. This precise targeting not only facilitates active tumor cell identification but also blocks the PD-1/PD-L1 immune checkpoint pathway, thereby exerting an immunotherapeutic effect. Concurrently, DSF increases intracellular Cu levels and promotes the aggregation of DLAT, as confirmed by Western blot (WB), ultimately leading to cuproptosis, and further enhancing treatment efficacy.

Metastasis is the leading cause of cancer-related deaths, accounting for approximately 90% [85]. An efficient and specific anti-metastasis strategy must effectively eliminate primary tumors while inducing systemic immunity to suppress distal metastasis [86]. Tumor metastasis systematically reprograms the microenvironment of distant organs and impairs immune cell function [87]. Therefore, eliminating metastatic foci requires strong systemic immune activation and durable immune memory. However, metastatic tumors often have a highly immunosuppressive TME, which limits the efficacy of most therapeutic strategies in eliciting systemic anti-tumor immune responses [88,89]. Thus, reshaping the immunosuppressive TME, such as promoting DC maturation and activating CD8^+^ T cells, is expected to enhance anti-tumor effects [90]. Studies have shown that cuproptosis not only directly kills tumor cells but also reshapes the TME, promoting DC maturation and immune cell infiltration [11]. For example, in a clear cell renal cell carcinoma model, elesclomol-Cu-induced cuproptosis activates the cGAS-STING pathway in DC, promoting the release of inflammatory mediators such as IFN-γ, TNF-α, IL-2, CXCL10, and CXCL11, significantly enhancing the anti-tumor immune response [91]. Yan et al. developed a biocompatible nanoplatform (Cu-HPB/C) that utilizes cavity confinement and porous matrix effects to encapsulate cholesterol oxidase within the hollow interior and mesoporous structure of Cu-HPB, achieving a drug loading rate of 86.75%. In an acidic environment, the released Cu^2+^ induces cuproptosis, while concurrently enhancing the activity of cholesterol oxidase, promoting cholesterol clearance and generating H_2_O_2_. Furthermore, Cu^2+^ catalyzes the Fenton-like reaction of H_2_O_2_ to produce highly toxic hydroxyl radicals (·OH), elevating oxidative stress levels and synergistically enhancing the efficacy of cuproptosis. In a 4T1 metastatic tumor model, the Cu-HPB/C treatment group showed extremely weak bioluminescence signals, demonstrating excellent anti-metastasis ability and significantly prolonging the survival of mice [92]. Although these studies demonstrate the great potential of cuproptosis in suppressing metastasis, the specific molecular mechanisms by which it reshapes the TME remain unclear and will be an important direction for future research. In summary, utilizing INPs to induce cuproptosis and enhance the therapeutic effects of tumor is a promising strategy.

## 3. Strategies for Synergistic Induction of Cuproptosis Based on INPs

Nanomedicine significantly enhances the efficacy of cancer therapy due to its unique advantages. First, ligand modification facilitates tumor-specific targeted delivery. Second, nanocarriers markedly improve drug stability and enhance the dissolution characteristics of poorly soluble drugs. In addition, optimizing the surface properties of nanocarriers can prolong the drug’s half-life. Moreover, the design of stimulus-responsive systems allows for TME-specific drug release. Furthermore, multifunctional carriers can deliver multiple therapeutic agents for synergistic therapy. Finally, nanocarriers can effectively navigate various biological barriers, including the blood–brain barrier [93,94]. INPs, as crucial carrier materials in nanomedicine, exhibit unique multifunctional properties. On the one hand, they possess exceptional optical, electromagnetic, acoustic, and catalytic characteristics, facilitating integrated diagnosis and therapy [95]. On the other hand, their high specific surface area and tunable pore structure create an ideal platform for efficient drug loading and controlled release [96]. More importantly, by precisely controlling the size, morphology, surface charge, and chemical composition of INPs, we can achieve targeted drug delivery, optimized distribution, and enhanced accumulation at target sites in vivo [97]. INPs provide multidimensional and customizable innovative strategies for efficiently inducing cuproptosis, enabling multimodal synergistic anti-tumor therapy based on cuproptosis. Strategies for inducing or enhancing cuproptosis primarily encompass four aspects, including improving Cu delivery efficiency, promoting the generation of Cu^+^, reducing Cu^2+^ chelation, and metabolic reprogramming.

### 3.1. Enhancing Cu Delivery Efficiency

Delivery efficiency can be significantly enhanced through increased drug loading, active and passive targeting strategies, and controlled release mechanisms. INPs such as mesoporous silica and hollow mesoporous Prussian blue (HMPB), owing to their high specific surface area and porous structures, enable efficient loading of Cu and its compounds (e.g., CuO, Cu_2_O) [98]. Meanwhile, they effectively protect Cu from premature release or degradation during delivery, ensuring high-concentration accumulation at the tumor site. Ma et al. constructed a Cu-based Prussian blue nanostructure (NCT-503@Cu-HMPB) loaded with a serine metabolism inhibitor (NCT-503), designed to achieve therapeutic effects through selective induction of cuproptosis and disruption of serine metabolism [99]. Cu directly induces abnormal aggregation of lipoylated TCA cycle proteins, thereby triggering cuproptosis. Meanwhile, NCT-503 impedes serine metabolism and severely disrupts GSH synthesis. This not only reduces chelation-mediated Cu sequestration but also further elevates cellular oxidative stress, significantly enhancing cuproptosis.

The advancement of drug delivery systems has provided a comprehensive “arsenal” for Cu-induced cuproptosis therapy, while targeted delivery serves as a crucial navigation mechanism for the precise management of “ammunition”. Achieving accurate delivery of therapeutic agents to tumor sites and attaining high concentrations of Cu accumulation within cancer cells remain one of the core challenges in evaluating the efficacy of cuproptosis. Through surface modification, nanoparticles can achieve tumor-specific accumulation via active or passive targeting strategies. By functionalizing the surface of inorganic nanomaterials—such as with MCF-7-specific aptamers, hyaluronic acid (HA), cRGDfk peptides, or cell membrane coating—active recognition and enrichment in tumor cells can be realized, enabling precise drug delivery and accumulation. Xu et al. developed a nanodrug for clear cell renal cell carcinoma (ccRCC) by encapsulating lactate oxidase (LOx) into a ccRCC cell membrane-camouflaged CuO@Gd_2_O_3_ yolk–shell-like particle (mCGYL-LOx) to activate cuproptosis [100]. The Renca renal cell membrane coating confers homologous targeting ability. The released LOx enzyme oxidizes excess lactate in ccRCC cells, generating pyruvate and H_2_O_2_, which enhances oxidative stress and further promotes cuproptosis. WB showed an increase in DLAT expression and a decrease in FDX1 expression, confirming the occurrence of cuproptosis.

INPs can achieve tumor-specific accumulation through active or passive targeting strategies. Passive targeting relies on particle size and surface hydrophilicity, such as polyethylene glycol (PEG) modification, facilitating tumor enrichment via the enhanced permeability and retention (EPR) effect [101,102]. Zhou et al. constructed a photothermal-responsive nanoplatform (Au@MSN-Cu/PEG/DSF) comprising a gold nanorod core and a mesoporous silica shell [103]. Surface PEGylation enhances the EPR effect, promoting efficient accumulation of nanoparticles in the tumor region. Under NIR (near-infrared) laser irradiation, the gold nanorod core generates a photothermal effect, and localized heating triggers the biodegradation of the Cu-doped silica framework, leading to controlled release of encapsulated Cu^2+^ and DSF for synergistic induction of cuproptosis.

The design of TME-responsive materials (e.g., to pH, GSH [104], H_2_S, or light) enables specific and intelligent drug release at the tumor site, significantly improving delivery efficiency and reducing systemic toxicity. Xu et al. developed a self-accelerating “Cu bomb” nanoplatform (CGNPs) based on Cu-doped mesoporous silica nanoparticles (MCNs) [105]. The low pH of the TME triggers the degradation of the sea urchin-like MCNs structure, resulting in the release of Cu^2+^ as a “Cu bomb”. Glucose oxidase (GOx) adsorbed on the MCNs surface consumes glucose to generate H_2_O_2_, further enhancing cuproptosis. Meanwhile, the gluconic acid produced during this process acidifies the local environment, forming a positive feedback loop that accelerates MCNs degradation and complete Cu^2+^ release, achieving a self-amplified drug release process. Given the significant overexpression of H_2_S of TME in the colorectal cancer, Zhao et al. designed an H_2_S-responsive Cu_2_(PO_4_)(OH) nanoparticle to induce cuproptosis [106]. The nanoparticles react with endogenous H_2_S in the colon cancer region, dissociating and transforming in situ into ultrasmall Cu_9_S_8_ nanoparticles. This process not only releases a large amount of Cu but also enhances intracellular ROS levels via Fenton-like reactions, thereby augmenting cuproptosis. Additionally, ATP7A expression is downregulated during this process, further reducing Cu efflux and promoting intracellular Cu accumulation.

### 3.2. Increasing Cu^+^ Generation

**Figure 3:**
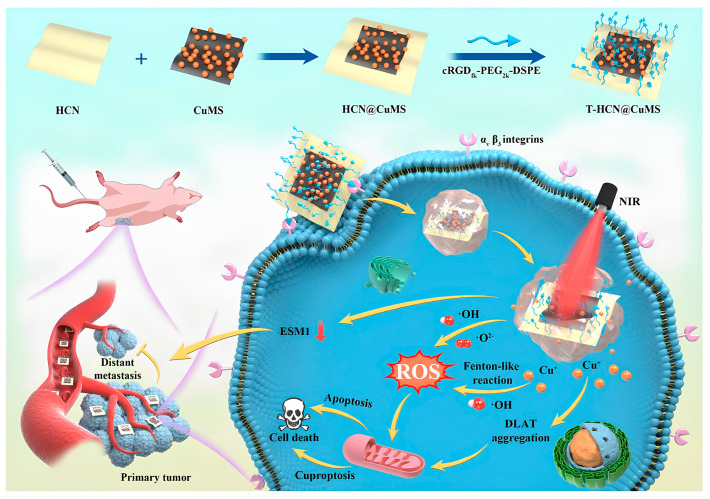
The cRGDfk-PEG_2_k-DSPE-modified T-HCN@CuMS nanoplatform enables αvβ_3_ integrin-targeted delivery. This system not only effectively anchors and reduces Cu^2+^ to Cu^+^ to induce cuproptosis but also generates substantial ROS under NIR laser irradiation. Furthermore, the NIR-activated T-HCN@CuMS significantly downregulates Endothelial cell-specific molecule 1 (ESM1), a secreted proteoglycan that promotes tumor metastasis. Reproduced with permission from [112].

Previous studies have shown that Cu^2+^ delivered into mitochondria can be reduced to Cu^+^ by FDX1, thereby enhancing cuproptosis. Thus, increasing the generation of Cu^+^ represents an effective strategy to potentiate cuproptosis. Research indicates DSF is reduced in vivo to its primary active metabolite, diethyldithiocarbamate (DDC). One DDC molecule can provide two sulfur atoms to form a stable cyclic chelation structure with a Cu. Two DDC molecules coordinate with one Cu to generate an electrically neutral Cu(DDC)_2_ complex. During the chelation process, the -SH group in DDC acts as a reducing agent, converting chelated Cu^2+^ into Cu^+^ [107,108]. Therefore, loading DSF onto INPs can effectively elevate mitochondrial Cu^+^ levels and enhance cuproptosis. Zhao et al. developed a novel nanotherapy named GOx-CuCaP-DSF, which was constructed by integrating GOx into calcium-doped calcium phosphate (CaP) nanoparticles with DSF adsorbed on the surface [109]. Within the TME, the released Cu^2+^ coordinates with DSF to form the highly cytotoxic chemotherapeutic drug and cuproptosis inducer—Cu(DDC)_2_ in situ. This reaction involving DSF elevates Cu^+^ levels, thereby enhancing cuproptosis. Furthermore, the H_2_O_2_ generated during the catalytic process of glucose oxidase, combined with mitochondrial dysfunction induced by Ca^2+^ overload, synergistically enhances the cuproptosis. Metallic transition metal dichalcogenides [110] and heterojunction-based composite nanomaterials [111] are capable of reducing Cu^2+^ to the more reactive Cu^+^. Xia et al. constructed a T-HCN@CuMS nano-heterojunction based on heterogeneous carbon nitride nanosheets (HCN) and Cu-loaded 1T-MoS_2_ nanosheets (CuMS) [112] (see Figure 3). CuMS not only serves as an efficient carrier for Cu but also reduces Cu^2+^ to Cu^+^, thereby inducing cuproptosis. The cRGDfk peptide modified on the surface enables specific recognition and binding to αvβ3 integrin, which is highly expressed on cancer cells, achieving active targeting. PEG modification further enhances the colloidal stability of the nanoparticles and prolongs their circulation time in vivo. Moreover, the multi-level heterostructure significantly improves the NIR-induced catalytic performance, generating substantial ROS and enhancing cellular sensitivity to cuproptosis. Cu_2_O serves as a natural reservoir of Cu^+^, providing the most direct source of Cu^+^ without requiring intracellular reduction. Huang et al. developed a novel ternary heterojunction nanomaterial named HACT (HA-modified TiO_2_ QDs/Au@Cu_2_O core–shell nanocubes) [113]. The HA component enables specific recognition and binding to CD44 receptors, promoting tumor accumulation and efficient internalization of the nanocomplex by cancer cells, thereby achieving targeted therapy. The released Cu^+^ not only strongly induces cuproptosis but also catalyzes the Fenton-like decomposition of H_2_O_2_ to generate highly toxic ·OH radicals. Meanwhile, the TiO_2_ quantum dots enhance sonodynamic therapy by improving the quantum yield under ultrasound irradiation, leading to substantial ROS generation. The ultrasound-triggered ROS synergize with ·OH produced via the Fenton-like reaction, collectively inducing severe intracellular oxidative stress and amplifying cuproptosis. Furthermore, the combined action of ultrasound and the TME allows precise activation and regulation of this process, significantly improving treatment safety and efficiency.

### 3.3. Reducing Cu^2+^ Chelation

GSH serves as a natural Cu chelator that binds Cu to mitigate cellular damage. Reducing intracellular GSH levels to elevate free Cu concentration represents an effective strategy for enhancing cuproptosis. Concurrently, GSH depletion further augments cellular oxidative stress, thereby synergistically amplifying the cuproptosis effect. Liu et al. developed a manganese-Cu co-loaded calcium carbonate nanoparticle system (CaCO_3_/Mn/Cu@lip-Apt) [114]. The high-valence Mn* effectively depletes intracellular GSH, enhancing cuproptosis. An aptamer conjugated to the nanoparticle surface enables specific recognition and binding to membrane proteins overexpressed on MCF-7 breast cancer cells, achieving active targeting. An outer lipid coating offers excellent biocompatibility and prolongs systemic circulation. The system employs a combined passive and active targeting strategy to improve therapeutic efficacy while minimizing side effects. Furthermore, under acidic conditions, released Ca^2+^ from the nanoparticles induce intracellular calcium overload, leading to mitochondrial dysfunction and significantly increasing cellular sensitivity to cuproptosis.

### 3.4. Metabolic Reprogramming

Tumor cells typically exhibit the Warburg effect, which is characterized by increased metabolic activity and dependence on glycolysis for energy supply [115,116]. Studies have reported that tumor cells reliant on glycolysis exhibit lower sensitivity to cuproptosis, making metabolic reprogramming an effective strategy for enhancing cuproptosis. Zu et al. developed a class of metabolically targeted Cu_2−x_S nanotherapeutic agents (MACuS) using a glucose-mediated biomineralization approach, employing glucose-6-phosphate (G6P) as a ligand, which can be specifically targeted to tumors via the GLUT-1 [117]. These nanoparticles can be preferentially internalized by tumor cells via glucose-mimicking metabolic uptake. The Cu-based nanomaterials supply sufficient Cu to effectively induce cuproptosis. Erastin (Er) potentiates tumor cell sensitivity to cuproptosis through its “anti-Warburg effect”. Li et al. developed a core–shell nanoparticle, CuP/Er, for the co-delivery of Cu and Er to cancer cells, enabling synergistic cuproptosis and ferroptosis [118]. This approach enhances sensitivity to cuproptosis by reducing tumor cell dependence on aerobic glycolysis, inhibiting System Xc^−^ function, and depleting GSH.

The induction of cuproptosis by INPs often involves synergistic multi-strategy approaches. For instance, Huang et al. developed a DSF-loaded hollow mesoporous Cu sulfide (HMCIS) nanoparticle (DSF@HMCIS-PEG-FA), modified with PEG and FA, which enables rapid release of DSF, H_2_S, Cu^2+^, and Fe^2+^ in the acidic TME [119]. The HMCIS enhances the loading efficiency of DSF, H_2_S, Cu^2+^, and Fe^2+^. Surface modification with PEG and FA promotes the accumulation of nanoparticles within tumor cells. DSF reduces Cu^2+^ to Cu^+^, thereby enhancing cuproptosis induction. Simultaneously, high-valence metal ions (Fe^3+^) deplete GSH, reducing Cu chelation and further potentiating the therapeutic effect. Multiple strategies synergistically induce cuproptosis, resembling the execution of a precise “burning plan” designed to trigger a lethal yet controllable fire inside cancer cells. In this process, Cu act as the “core fuel”, directly triggering cuproptosis. INPs serve as the “advanced combustion chambers”, enabling efficient loading and transport of the fuel. Targeted delivery systems function like “precision guidance”, ensuring the combustion chambers are accurately delivered to the cancer cell “headquarters”. The responsive release mechanism works like an “intelligent detonator”, activating the release of the fuel in response to specific environmental signals. Inhibiting Cu^2+^ chelation is akin to disabling the cancer cell’s own “firefighting system”. Meanwhile, metabolic reprogramming alters the “terrain and wind direction” of the cancer cell, forcing it into a more easily ignitable metabolic state. Furthermore, several “combustion-boosting” strategies—such as inducing mitochondrial dysfunction and DNA damage through ROS, calcium ions [114], or prodigiosin [120]—ultimately fuel the flames of cuproptosis and eliminate tumor cells. The application of INPs to induce cuproptosis is shown in Table 1.

## 4. Synergistic Cuproptosis-Multimodal Antitumor Therapy Mediated by INPs

### 4.1. Cuproptosis Combined with IT

**Figure 4:**
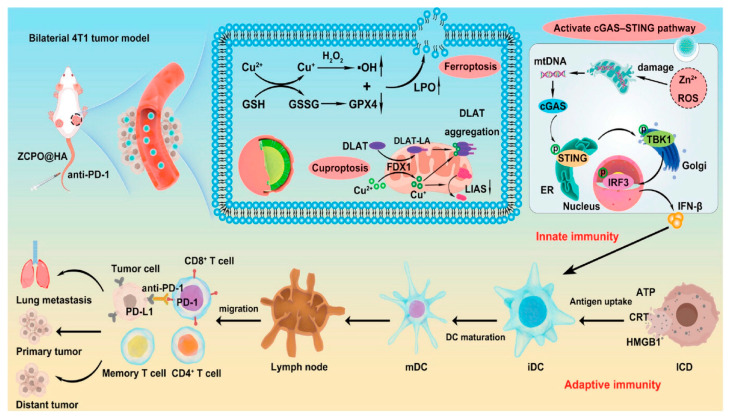
HA-modified zinc-copper bimetallic peroxide (ZCPO@HA) nanoparticles release Zn^2+^ and Cu^2+^ in the weakly acidic TME, effectively inducing both ferroptosis and cuproptosis. The resulting ICD releases damage-associated molecular patterns (DAMPs), activating adaptive antitumor immunity. Concurrently, mitochondrial damage promotes the release of mitochondrial DNA, which activates the cGAS-STING pathway and initiates innate immune responses. With the assistance of anti-PD-1, these nanoparticles leverage this synergistic immunotherapeutic effect to potently suppress tumor growth and metastasis. Reproduced with permission from [121].

The cancer therapy strategy has evolved from traditional surgery, RT and chemotherapy [144] to a new paradigm of multimodal combination therapy, in which the advent of IT has revolutionized the clinical treatment landscape [145] Cuproptosis is a novel form of cell death that can promote immune cell infiltration [19], providing an innovative breakthrough for enhancing IT efficacy. Liu et al. synthesized HA-modified zinc-Cu bimetallic peroxide (ZCPO@HA) nanoparticles via a one-step symbiotic method [121] (see Figure 4). This material induces ferroptosis and cuproptosis through Fenton-like reactions in TME, while activating the cGAS-STING pathway to enhance innate immunity, and achieves efficient IT in combination with immune checkpoint inhibitors. Xie et al. developed Cu_2_O-MnO@PEG (CMP) nanomaterials, which directly induce cuproptosis in tumor cells and activate anti-tumor immune responses (enhancing antigen presentation, promoting CD8^+^ T cell responses, and inhibiting Treg cells), achieving tumor suppression and establishing long-term immune memory in combination with PD-L1 monoclonal antibodies, showing significant clinical translation potential [122]. The hypoxic TME not only hinders the efficacy of IT [146], but also suppresses cuproptosis [147]. Tao et al. developed oxygen pump microneedles (OPMNs-ZCS@siPD-L1) loaded with zinc-doped Cu sulfide nanoflowers (ZCS NFs) and PD-L1 siRNA. Through a “dual-effect synergy” strategy—“accelerating” [cuproptosis activates the STING pathway/induces immunogenic cell death (ICD)] and “releasing the brake” (PD-L1 inhibition/improving hypoxia)—they significantly enhance IT efficacy and inhibit metastasis [123]. Cu ionophores combined with immune agonists form an effective combination therapy. Zhao et al. designed a Cu sulfide-DSF nanocomposite (DSF/CuS-C) based on the Toll-like receptor agonist CpG template. By reprogramming TME and inducing cuproptosis in synergy with ICD, they achieved efficient breast cancer treatment in mouse models [124]. Lu et al. constructed an intelligent nanoplatform (Es@CuO) with Es-encapsulated Cu oxide nanoparticles (CuO). By synergistically releasing Cu^2+^ and Es to induce cuproptosis, they significantly inhibit melanoma growth and enhance anti-tumor effects when combined with PD-1 inhibition [125]. Recent studies have shown that the levels of Cu [148] and metabolism [149] in tumors are critical for regulating PD-L1 expression. Xu et al. developed a lung cancer cell membrane-coated GOx-Cu-LDH biomimetic nanodelivery system (CMGCL). By interfering with glucose and Cu metabolism, it synergistically upregulates PD-L1 expression and induces cuproptosis. The system showed excellent targeting and biosafety in a lung cancer model, and combined with αPD-L1, it significantly enhanced anti-tumor effects, providing a new strategy for combining metabolic intervention, cuproptosis, and immunotherapy [126]. The synergistic effect of cuproptosis and IT opens new avenues for cancer therapy.

### 4.2. Cuproptosis Combined with PTT

**Figure 5:**
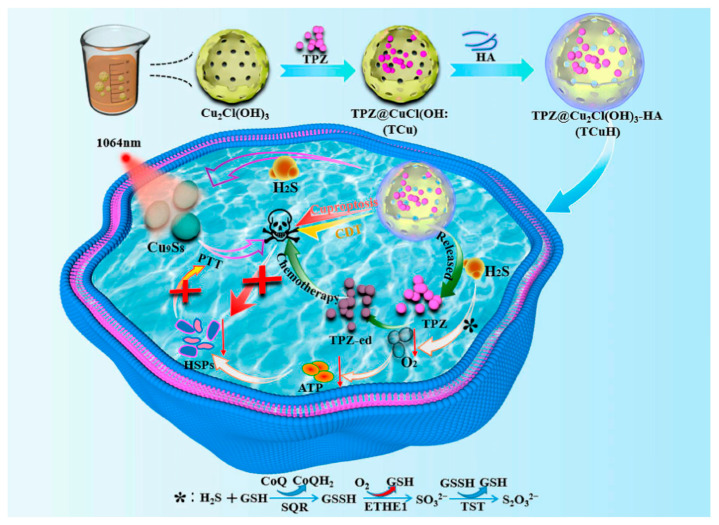
Upon activation by excess H_2_S and NIR-II light in TME, HA-modified TCuH nanoparticles generate Cu_9_S_8_ while releasing the hypoxia-activated prodrug TPZ. The activated TPZ exerts chemotherapeutic effects, concurrently with enhanced mild PTT efficacy due to reduced heat shock protein expression. Furthermore, the enriched Cu subsequently induces cuproptosis, ultimately synergizing to elicit multifaceted antitumor effects. Reproduced with permission from [127].

PTT, as an emerging tumor therapy strategy, utilizes photothermal agents to convert light energy into heat energy under NIR light irradiation, precisely killing tumor cells with advantages such as strong spatiotemporal controllability, minimal invasiveness, and fewer side effects [150,151]. Among various photothermal agents, INPs have attracted much attention due to their excellent photothermal conversion efficiency and stability, including noble metal nanomaterials [152,153], metal sulfides [154], and two-dimensional materials [155,156]. Cu-based sulfides (e.g., Cu_2−x_S, Cu_2−x_Se) have become efficient photothermal agents for deep tumor therapy due to their unique plasmon resonance effect and NIR-II region absorption characteristics [157,158,159,160,161]. For instance, Qiao et al. constructed H_2_S-responsive TPZ@Cu_2_Cl(OH)_3_-HA (TCuH) that achieves PTT by triggering in situ generation of Cu_9_S_8_, while coupling H_2_S consumption-induced mitochondrial reprogramming to activate hypoxic prodrug tirapazamine (TPZ) for chemotherapy, cuproptosis, and chemodynamic therapy, utilizing the high H_2_S TME in colon cancer to achieve a quadruple synergistic anti-tumor mechanism [127] (see Figure 5). In vivo evaluation in a CT26 subcutaneous tumor model demonstrated that TCuH plus laser irradiation achieved a remarkable tumor inhibition rate of 92.3%. This potent efficacy was coupled with mechanistic validation; immunofluorescence confirmed the downregulation of FDX1 in tumors, corroborating the induction of cuproptosis as a key cell death pathway. Similarly, Cheng et al. proposed a self-amplifying nanoplatform (CEL NP) based on Cu_2−x_S hollow nanospheres (HNSs). This platform enhances cuproptosis and ICD in colon cancer by triggering Es and Cu release via NIR-II activation [128]. In a CT26 tumor model, HNSs plus laser irradiation achieved potent tumor growth inhibition and prolonged survival. Mechanistic studies confirmed cuproptosis induction via downregulation of FDX1, LIAS, and ATP7B by WB, and robust antitumor immunity evidenced by increased infiltration of DC, CD^3+^, and CD8^+^ T cells. Cu_2−x_Se is an ideal thermoelectric material, and its Cu vacancy-triggered plasmon absorption lies in the NIR-II region, which has centimeter-level tissue penetration and reduced light scattering [162,163,164,165]. Yang et al. proposed Cu_2−x_Se HNSs for plasma thermoelectric catalytic therapy, utilizing NIR-II laser-triggered photothermal conversion to generate local temperature gradients, driving thermoelectric catalytic ROS production and cuproptosis, achieving synergistic anticancer effects via mitochondrial dysfunction through two pathways [129]. The combination therapy mediated near-complete tumor eradication in CT26 models, which was mechanistically supported by WB confirmed FDX1/LIAS downregulation and DLAT upregulation, alongside transmission electron microscopy-observed severe mitochondrial damage—hallmarks of potent cuproptosis induction.

In recent years, photothermal-driven nanomotors have shown great potential in cancer therapy due to their unique autonomous motion characteristics and excellent tumor penetration ability. Song et al. developed NIR light-driven nanomotors (CuSiO_3_@Au-Pd NMs) for cuproptosis-assisted synergistic therapy of breast cancer. CuSiO_3_@Au-Pd nanomotors exhibited 33% photothermal efficiency and unique self-propulsion that enhanced deep tumor penetration, as validated in 3D spheroid models. This resulted in remarkable anticancer efficacy in vivo, mechanistically linked to cuproptosis induction via FDX1 downregulation and DLAT oligomerization (WB-confirmed) [130]. At the same time, significant progress has been made in the research of nanozymes based on multivalent metal ions (Cu^1+/2+^, Fe^2+/3+^, Mn^2+/4+^) catalytic properties. These materials can precisely regulate TME by mimicking the activities of glutathione oxidase, peroxidase, and catalase, significantly enhancing the therapeutic effects [166,167,168]. The core–shell structured CMCO nanom enzyme designed by Chen’s team innovatively utilizes Cu ionophores activated by TME, synergistically enhancing catalytic activity through photothermal effects, offering new insights into cuproptosis [131]. To investigate cell death mechanisms, inhibitors of apoptosis (Z-VAD-FMK) and ferroptosis (Ferrostatin-1), along with FDX1 siRNA, were used. In CT26 cells treated with CMCO and 1064 nm laser, cuproptosis (50.9%) was the predominant cell death mechanism, followed by apoptosis (10.3%) and ferroptosis (9.7%). WB showed reduced FDX1 and elevated DLAT expression. FDX1 siRNA restored cell viability. In a CT26 tumor model, the combination treatment group showed significant tumor growth inhibition and superior antitumor efficacy. Furthermore, the development of new nanom enzyme systems such as CuMoO_4_ [132], Cu_5.4_O [133], CuSACO [134], Cu_9_S_8_ [135], and Cu-BiSex [136] has further expanded the scope of PTT. Notably, the GSH/pH dual-responsive MCD nanoparticles developed by Ye et al. target the disruption of tumor energy metabolism through the synergistic effect of cuproptosis and ROS, combined with NIR-II precise PTT, demonstrating unique advantages in inhibiting osteosarcoma growth and protecting bone tissue [137]. The MCD-laser combination potently suppressed cell viability (~30%) in vitro and significantly inhibited orthotopic osteosarcoma growth and metastasis in vivo, as shown by minimal tumor burden, lowest Ki67^+^ rate (18.7%), and best antimetastatic effect by imaging. This efficacy was mechanistically linked to cuproptosis induction, evidenced by WB-confirmed downregulation of FDX1 and LIAS. These studies provide important insights into the combination of PTT and cuproptosis.

### 4.3. Cuproptosis Combined with Sonodynamic Therapy

**Figure 6:**
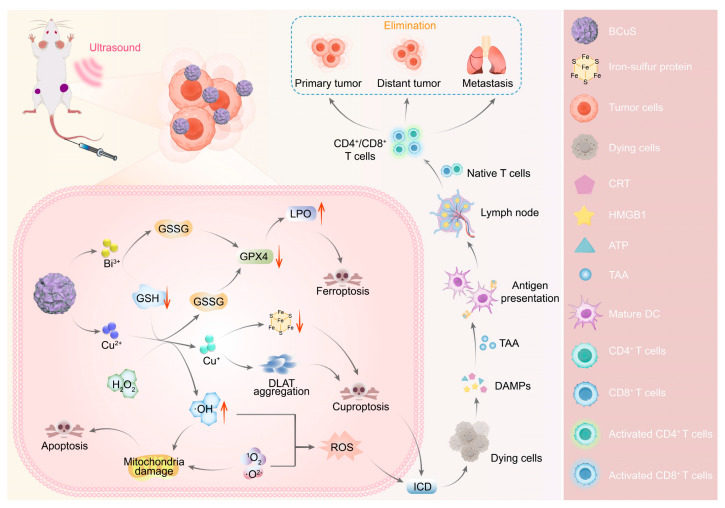
The BCuS nanoparticles with p-n heterojunction generate ROS under ultrasound stimulation to induce oxidative stress, and release Cu^2+^ and Bi^3+^ in the presence of GSH. Cu^2+^ is converted into highly toxic Cu^+^ via a Fenton-like reaction to induce cuproptosis, while the simultaneously produced ·OH synergizes with Bi^3+^-mediated GSH depletion to inactivate glutathione peroxidase 4, triggering ferroptosis. The combined effect of ferroptosis and cuproptosis activates ICD, which promotes the release of DAMPs to facilitate DC maturation and T-cell activation, ultimately leading to significant suppression of both local and distant tumor growth. Reproduced with permission from [138].

Sonodynamic therapy is a non-invasive therapeutic strategy based on ROS, offering deep tissue penetration and minimal invasiveness, and has been widely studied for tumor therapy [169,170,171]. However, most organic sonosensitizers are inevitably limited by their stability and harmful phototoxicity, which greatly restricts their biological applications [172,173]. Recently, various inorganic sonosensitizers with controllable physicochemical properties, high chemical stability, and favorable pharmacokinetic characteristics have emerged, such as Ti_3_C_2_/CuO_2_ nanosheets [174], MnWOx nanoparticles [175], MoS_2_ nanosheets [176], and TiO_1+x_ nanorods [177]. However, the rapid recombination of ultrasound-triggered electrons (e^−^) and holes (h^+^) limits ROS production [178,179]. In recent years, the construction of heterojunctions with matching band gaps has been explored to suppress the recombination of electron-hole pairs [180,181]. At the same time, the addition of Cu^2+^ not only consumes GSH, reducing ROS consumption and enhancing sonodynamic therapy, but the reduced Cu^1+^ has better cuproptosis effects. Wu et al. adsorbed the p-type semiconductor CuS onto the surface of the n-type semiconductor Bi_2_O_3−X_S_X_, constructing a Bi_2_O_3−X_S_X_-CuS (BCuS) p-n heterojunction [138] (see Figure 6). Notably, the p-n heterojunction facilitates the rapid separation of electron-hole pairs while inhibiting their recombination, representing an advanced strategy. BCuS produced a large amount of ROS during US stimulation, and Cu^2+^, Bi^3+^, and holes consumed GSH, inducing cuproptosis, promoting the maturation of DC and triggering systemic immune responses. This novel p-n heterojunction Cu-based sonosensitizer provides a direction for sonodynamic therapy combined with cuproptosis to enhance IT. In a 4T1 subcutaneous tumor model, BCuS combined with ultrasound achieved a 92.3% tumor inhibition rate—significantly higher than BCuS alone (48.0%). Immunohistochemistry showed enhanced DLAT aggregation, confirming cuproptosis activation. Flow cytometry revealed increased CD8^+^ T cells (28.9% to 37.4%) and enhanced DC maturation, indicating robust antitumor immunity. Cheng et al. also constructed Cu-doped carbon dot p-n type semiconductors (Cu-CDs), improving the electron-hole separation efficiency, increasing ROS production, and enhancing sonodynamic therapy. Notably, Cu-CDs also have good blood–brain barrier permeability and strong antitumor activity, making them suitable for the treatment of glioblastoma multiforme [139]. In an orthotopic U87-Luc glioma model, Cu-CDs plus ultrasound treatment markedly suppressed tumor growth and resulted in a near 100% survival rate. Immunofluorescence confirmed upregulated DLAT and downregulated FDX1/LIAS, indicating effective cuproptosis activation. Yan et al. constructed a Z-type heterojunction (GQD/Cu_2_O) using graphene QDs and Cu_2_O, with Cu^2+^ as a transition metal enhancing the Jahn-Teller effect to increase sonodynamic therapy efficacy [140]. WB revealed that combined treatment downregulated FDX1 and LIAS while upregulating DLAT. In a bilateral 4T1 model, GQD/Cu_2_O plus ultrasound eradicated primary tumors and potently inhibited distant growth within 6 days. Flow cytometry confirmed enhanced DC maturation and activation of CD4^+^/CD8^+^ T cells, indicating robust immune stimulation. Tang et al. developed Cu-substituted ZnAl ternary layered double hydroxide nanosheets as sonosensitizers and Cu nanocarriers. The introduction of Cu^2+^ into ZnAl nanosheets triggered a strong Jahn-Teller effect, accompanied by lattice distortion and atomic disorder, generating numerous defects (such as vacancies, dislocations), increasing active sites, which facilitated ROS generation and enhanced sonodynamic therapy performance [141]. Meanwhile, Cu^2+^ reacts with GSH, and the generated Cu^+^ leads to the abnormal oligomerization of acylated proteins, triggering synergistic sonodynamic therapy/cuproptosis. ZCA plus ultrasound eradicated CT26 subcutaneous tumors within 4 days. In a 4T1-luc breast cancer model, the combination completely suppressed tumor growth and metastasis, achieving 100% survival over 60 days. Notably, cuproptosis combined with sonodynamic therapy also enhances the immunogenic response of tumors, making it a promising approach for tumor treatment.

### 4.4. Cuproptosis Combined with RT

RT, as an important method for cancer therapy, is used for the cure or palliative treatment of localized tumors or oligometastatic tumors, with more than 50% of cancer patients undergoing RT [182,183,184]. However, repeated RT leads to the development of radiation resistance in residual tumors [185]. The radiation resistance of tumors increases the risk of local recurrence, and re-irradiation requires increased radiation doses to achieve the previous effect [186]. However, normal tissues have limited tolerance to radiation doses, which limits high-dose RT. Although precision RT techniques have achieved great success, radiation sensitization remains an essential strategy. A novel radiation sensitization strategy based on INPs inducing cuproptosis holds promise in addressing the clinical challenges of radiation resistance. To study the cuproptosis related characteristics in residual tumors, Liao et al. evaluated the expression of Cu-related features in human cervical cancer tumors and CT26 mouse tumors after fractionated RT. They found that FDX1 and LIAS were upregulated in the tumors, and played key roles in promoting cuproptosis [142]. Notably, radiation-resistant cells exhibit higher susceptibility to cuproptosis compared to their parental cells. Next, they synthesized Cu-containing polyoxometalate PWCu by reacting CuCl_2_·2H_2_O and heat-treated Na_8_HPW_9_O_34_·19H_2_O (PW_9_) in a mildly heated aqueous solution [142]. The structure of PWCu resembles a capsule, with both ends consisting of [(PW_9_O_34_)_2_Cu_4_(H_2_O)_2_]^10−^ polyoxoanion made of two B-α-isomeric PW_9_ polyoxoanions, with four coplanar CuO_6_ octahedra in the middle, displaying ultrasmall particles (1–1.6 nm). PWCu nanocapsules are highly soluble in water and can be fully exposed to TME, releasing cytotoxic Cu^+^ in a radiation-controlled manner within cancer cells, thereby achieving radiation sensitization. Furthermore, PWCu-mediated radiation sensitization can effectively trigger immunogenic cuproptosis and increase the levels of DAMPs in the treated tumors. Using a bilateral 4T1-R tumor model, local injection of PWCu nanocapsules combined with low-dose radiation (IR) completely eliminated both primary and distant tumors. This combination significantly upregulated FDX1 and LIAS expression, enhanced DLAT aggregation and DAMP release, and markedly increased infiltration of CD4^+^ T cells (4.14-fold) and interferon-γ^+^CD8^+^ T cells (6.25-fold) in distant tumors compared to IR alone. In recurrent 4T1 tumors after initial irradiation, the combined treatment extended median survival from 56 days (monotherapy) to 90 days, achieving 40% complete remission. This study provides initial insights into the cell death-related characteristics of radiation resistance and offers a new perspective for re-irradiation sensitization.

**Figure 7:**
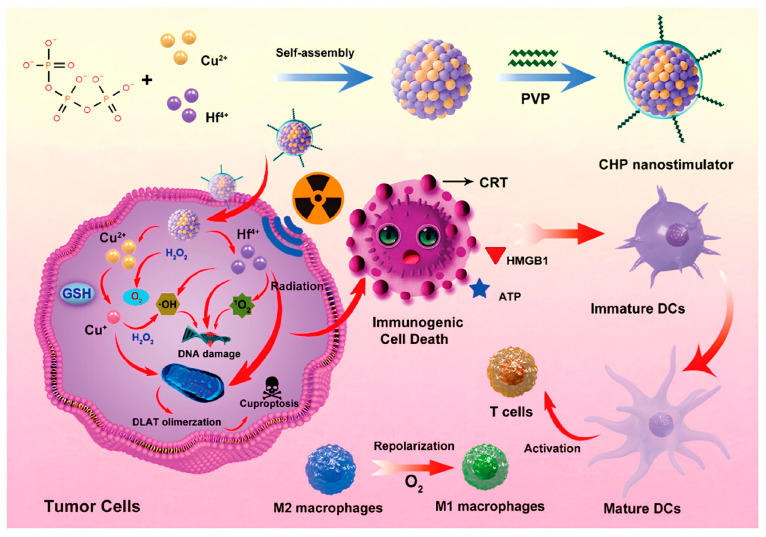
The bimetallic hybrid nanoregulator constructed through phosphate backbone coordination of Cu^2+^ and Hf^4+^ can be specifically activated within the acidic tumor microenvironment. The subsequently released Hf^4+^ interact physically with X-rays, triggering electron release and generating ROS, ultimately leading to irreparable double-strand DNA breaks and tumor cell apoptosis. Simultaneously released Cu induces cascaded amplification of oxidative stress by depleting GSH while effectively triggering cuproptosis. These synergistic mechanisms collectively enhance ICD, thereby successfully activating a systemic antitumor immune response under low-dose radiotherapy conditions. Reproduced with permission from [143].

The ICD strategy induced by RT helps address the limitations of RT, which is only used for local tumor control [187,188]. X-ray-induced ICD leads to the exposure or release of DAMPs, which can activate immune cells [189,190]. However, radiation dose dependence, low immunogenicity, and the complex and immunosuppressive TME pose significant challenges to the effective activation of radiation-induced ICD [191,192,193]. To address the high GSH levels, excessive H_2_O_2_, hypoxia, and the infiltration of numerous M2 phenotype tumor-associated macrophages in TME, Jiang et al. doped Cu and hafnium ions into a phosphate scaffold provided by sodium tripolyphosphate. Due to electronegativity differences, they self-assembled a bimetallic mixed nanostimulator, which was then surface-modified with polyvinylpyrrolidone (CHP) [143] (see Figure 7). CHP contains metal-phosphate bonds that can be responsively activated in acidic TME. The activation leads to GSH depletion, oxygen generation, and hydroxyl radical production, which promotes DC maturation and enhances the polarization of macrophages from the M2 phenotype to the M1 phenotype. Simultaneously, Cu binds with DLAT to induce cuproptosis in tumor cells, and these mechanisms collectively enhance ICD. Notably, the study found that the aggregation of DLAT was enhanced after exposure to X-ray radiation, indicating that radiation may facilitate the amplification of cuproptosis. In a bilateral TNBC model, CHP combined with low-dose (2 Gy) X-ray irradiation significantly inhibited tumor growth, achieving suppression rates of 82.1% (primary) and 77.0% (distant) within 20 days. The treatment also promoted DC maturation and enhanced activation of CD4^+^ and CD8^+^ T cells, stimulating antitumor immunity. Overall, cuproptosis induced by INPs to enhance RT sensitivity is a promising tumor therapy approach, expected to solve the clinical challenges of radiation resistance. Furthermore, in this process, RT may also amplify the effects of cuproptosis, providing valuable insights for the expansion of the cuproptosis mechanism.

## 5. Challenges and Future Perspectives

Cuproptosis has been applied in various fields including cancer therapy, antibacterial applications, and Alzheimer’s disease [194,195,196,197]. INPs, leveraging their exceptional drug-loading capacity and high modifiability, can effectively address the issues of insufficient Cu accumulation and poor targeting, thereby efficiently inducing cuproptosis. In addition, due to their unique physicochemical properties, they can be developed as photothermal agents, photosensitizers, and sonosensitizers, widely applied in combination therapies for cancer, thus expanding treatment strategies. More importantly, through special structures such as heterojunctions [138] and Jahn-Teller effects [141], electron-hole recombination can be suppressed, increasing the active sites for ROS generation, thus enhancing oxidative stress response and further improving therapeutic efficacy. In conclusion, significant progress has been made in research on inducing cuproptosis using INPs, in the field of tumor therapy.

Despite the significant advantages of INPs in inducing cuproptosis, they still face multiple challenges. First, the biocompatibility of nanomaterials that induce cuproptosis is a major challenge [198], as excessive Cu and the nanomaterials themselves may have adverse effects on normal cells and organisms. This requires INPs to achieve precise delivery, biodegradability, as well as assess pharmacokinetics and monitor Cu concentrations in non-target organs. Secondly, there are differences in TME between different types of tumors [199], such as pH, fibrosis, immune responses, and hypoxia, which pose challenges for the development of INPs. Therefore, there is a need to design personalized INPs based on the tumor types. Finally, tumor metabolic heterogeneity (such as the Warburg effect) requires INPs to integrate metabolic reprogramming functions, such as co-delivery of HK_2_ inhibitors [200], to force glycolysis to switch to oxidative phosphorylation, enhancing cuproptosis sensitivity. In the face of these challenges, researchers need to have interdisciplinary collaboration skills, integrating knowledge of material science, tumor biology, and clinical medicine to explore the dynamic interaction mechanisms between cuproptosis and TME. At the same time, the safety and efficacy of nanomaterials systems must be rigorously evaluated to promote full-chain innovation from basic research to clinical translation, ultimately achieving breakthrough applications of cuproptosis in precision tumor therapy.

Engineering INPs to induce cuproptosis for cancer therapy is a promising strategy. The following are some insights into the progress of this research: (1) Design INPs based on TME characteristics, such as pH/GSH/H_2_S-responsive release, hypoxia [hypoxia-activated prodrug TPZ [127] or inhibition of HIF-1α [201]], and cold tumors [STING agonists increasing immune infiltration [123]]. (2) Integrate MALDI mass spectrometry imaging (spatial metabolomics) with single-cell transcriptomics (scRNA-seq) to map the subcellular localization of acylated proteins (such as DLAT, FDX1) and establish a cuproptosis sensitivity prediction model. (3) Cu concentration is a critical factor in maintaining normal cellular function, and even minor fluctuations can significantly impact cell viability. Therefore, there is an urgent need for advanced Cu monitoring technologies to enable real-time tracking of Cu levels during treatment. (4) Targeted drug delivery based on the molecular expression characteristics of tumor cells, such as high expression of PD-L1, HER2. (5) Conduct studies on the long-term biocompatibility of INPs for inducing cuproptosis in cells and organisms. The future clinical application prospects of INPs to induce cuproptosis for cancer therapy are vast, requiring continuous research.

## 6. Conclusions

INP-based strategies for inducing cuproptosis offer a novel approach for cancer treatment. Through surface functionalization and precise regulation of intracellular Cu metabolism, INPs can effectively address key challenges such as insufficient Cu accumulation in tumor tissues, systemic toxicity, and the lack of specific carriers, thereby efficiently triggering cuproptosis and enabling multimodal synergistic therapy. Although significant progress has been made in material design, mechanistic exploration, and combination therapies, further optimization of biocompatibility, TME adaptability, and metabolic reprogramming capabilities is still required. Future efforts should focus on addressing the current challenges and advancing the promising directions outlined above to ultimately promote the broad application of cuproptosis in precision cancer therapy.

## Figures and Tables

**Table 1:** Summary of the application of INPs to induce cuproptosis.

Nanomaterials	Route of Administration	Chemical Modulators	Targeting Mechanism	Response	Enhancement Strategy	Disease	Reference
CuX-P	Iv ^a^	Cu^2+^/Cu^+^/DSF	T cell membrane	-	Cu^+^ increase	Breast cancer	[84]
Cu-HPB/C	Iv	Cu^2+^/ChOx	EPR	PH	ChOx, ROS increase, and Cu^2+^ chelation decrease	Breast cancer	[92]
NCT-503@Cu-HMPB	Iv	Cu^2+^/NCT-503	EPR	PH/GSH	Disrupting serine metabolism, ROS increase, and Cu^2+^ chelation decrease	Breast cancer	[99]
mCGYL-LOx	Iv	Cu^2+^/LOx	Renca cell membrane	-	Lox, ROS increase, and Cu^2+^ chelation decrease	Kidney cancer	[100]
Au@MSN-Cu/PEG/DSF	Iv	Cu^2+^/Cu^+^/DSF	EPR	NIR-II	Cu^+^ increase	Breast cancer	[103]
CGNPs	Iv	Cu^2+^/GOx	tLyp-1 peptide and EPR	PH	GOx, metabolic reprogramming, ROS increase, and Cu^2+^ chelation decrease	Breast cancer	[105]
Cu_2_(PO_4_)(OH)	Iv	Cu^2+^/Cu^+^	EPR	H_2_S	Cu^+^ increase	Colon cancer	[106]
GOx-CuCaP-DSF	Iv	Cu^2+^/Cu^+^/DSF/GOx	EPR	PH	GOx, metabolic reprogramming, ROS increase, Cu^2+^ chelation decrease, Cu^+^ increase, and calcium overload	Hepatocarcinoma	[109]
T-HCN@CuMS	Iv	Cu^2+^	cRGDfk and EPR	-	Heterojunction, ROS increase, and Cu^2+^ chelation decrease	Sarcoma of bone	[112]
HACT	Iv	Cu^2+^	HA	PH/US	Heterojunction, ROS increase, and Cu^2+^ chelation decrease	Melanoma	[113]
CaCO_3_/Mn/Cu@lip-Apt	Iv	Cu^2+^/Mn*/Ca^2+^	MCF-7-specific aptamer and EPR	PH	ROS increase, Cu^2+^ chelation decrease, and calcium overload	Breast cancer	[114]
MACuS	Iv	Cu^2+^	GLUT-1	-	Metabolic reprogramming	Breast cancer	[117]
CuP/Er	Iv	Cu^2+^/Er	EPR	PH	Er, metabolic reprogramming, ROS increase, and Cu^2+^ chelation decrease	Colon cancer/Breast cancer	[118]
DSF@HMCIS-PEG-FA	Iv	Cu^2+^/Cu^+^/DSF/Fe^2+^	FA	PH	H_2_S, ROS increase, Cu^2+^ chelation decrease, and Cu^+^ increased	Breast cancer/Gastric cancer	[119]
ZCPO@HA	Iv	Cu^2+^/Zn^2+^	HA	PH	ROS increase, and Cu^2+^ chelation decrease	Breast cancer	[121]
Cu_2_O-MnO@PEG	Iv	Cu^2+^/Cu^+^	EPR	PH	Cu^+^ increase	Melanoma	[122]
OPMNs-ZCS@siPD-L1	Microneedle patch	Cu^2+^	Microneedle patch	PH	-	Melanoma	[123]
DSF/CuS-C	Iv	Cu^2+^/Cu^+^/DSF/CpG	EPR	TME	Cu^+^ increase	Breast cancer	[124]
ES@CuO	Iv	Cu^2+^/Cu^+^/Es	EPR	PH	Es	Melanoma	[125]
CMGCL	Iv	Cu^2+^/GOx	LLC membrane	PH	Gox, metabolic reprogramming, ROS increase, and Cu^2+^ chelation decrease	Lung cancer	[126]
TCuH	Iv	Cu^2+^/TPZ	HA and EPR	H_2_S	-	Colon cancer	[127]
CEL NP	Iv	Cu^2+^/Es	EPR	NIR-II/PH	Es	Colon cancer	[128]
Cu_2−x_Se HNSs	Iv	Cu^2+^	EPR	NIR-II	Thermoelectrocatalysis, ROS increase, and Cu^2+^ chelation decrease	Colon cancer	[129]
CuSiO_3_@Au-Pd NMs	Iv	Cu^2+^	EPR	PH	-	Breast cancer	[130]
CMCO	Iv	Cu^2+^/Cu_+_	EPR	TME	Nanozyme, ROS increase, Cu^2+^ chelation decrease, and Cu^+^ increase	Colon cancer	[131]
CuMoO^4^	Iv	Cu^2+^	EPR	PH	Nanozyme, ROS increase, and Cu^2+^ chelation decrease	Breast cancer	[132]
Cu_5.4_O	Oral administration	Cu^2+^/Cu^+^	EPR	-	Nanozyme, ROS increase, Cu^2+^ chelation decrease, and Cu^+^ increase	Colon cancer	[133]
CuSACO	Iv	Cu^2+^	EPR	GSH	Nanozyme, ROS increase, and Cu^2+^ chelation decrease	Breast cancer	[134]
CDCuCDs	Iv	Cu^2+^/Cu^+^	EPR	H_2_S	H_2_S, ROS increase, Cu^2+^ chelation decrease, and Cu^+^ increased	Colon cancer	[135]
Cu-BiSex	Iv	Cu^2+^	EPR	-	Nanozyme, ROS increase, and Cu^2+^ chelation decrease	Prostate cancer	[136]
MCD	Iv	Cu^2+^/Cu^+^	EPR	PH/GSH	ROS increase, Cu^2+^ chelation decrease, and Cu^+^ increase	Sarcoma of bone	[137]
BCuS	Iv	Cu^2+^	EPR	TME/PH	Heterojunction, ROS increase, and Cu^2+^ chelation decrease	Breast cancer	[138]
Cu-CDs	Iv	Cu^2+^	EPR	US	Heterojunction, ROS increase, and Cu^2+^ chelation decrease	Glioblastoma	[139]
GQD/Cu_2_O	Iv	Cu^2+^	EPR	PH	Heterojunction, ROS increase, and Cu^2+^ chelation decrease	Breast cancer	[140]
ZCA NSs	intratumoral injection	Cu^2+^	EPR	US	Jahn-Teller effect, ROS increase, and Cu^2+^ chelation decrease	Colon cancer/Breast cancer	[141]
PWCu	Iv	Cu^2+^/Cu^+^	EPR	X-Ray	X-rays, FDX1 and LIAS expression upregulated, and Cu^+^ increased	Breast cancer	[142]
CHP	Iv	Cu^2+^/Cu^+^/Hf^4+^	EPR	TME/PH	X-rays, ROS increase, and Cu^2+^ chelation decrease	Breast cancer	[143]

^a^ Intravenous injection.
